# Real‐World Diagnostic Workup of Patients Suspected for Light Chain Amyloidosis and Wild‐Type Transthyretin Amyloid Cardiomyopathy: A Retrospective Cohort Study Using US Electronic Health Records

**DOI:** 10.1002/jha2.70330

**Published:** 2026-06-15

**Authors:** Muhamed Baljevic, Haechung Chung, Jose Alvir, Patrick Feron, Aaron Crowley, Darrin Benjumea, Yong Chen, Cindi Pankratova

**Affiliations:** ^1^ Vanderbilt University Medical Center Nashville Tennessee USA; ^2^ Pfizer Inc New York New York USA; ^3^ Genesis Research Hoboken New Jersey USA; ^4^ Pfizer Inc Collegeville Pennsylvania USA

**Keywords:** AL amyloidosis, ATTR‐CM, cardiomyopathy, diagnosis, testing

## Abstract

**Introduction:**

Light chain (AL) amyloidosis and transthyretin amyloid cardiomyopathy (ATTR‐CM) are the most common types of cardiac amyloidosis. Despite similar manifestations, prognosis and treatments are distinct, emphasizing the importance of accurate and timely diagnosis. This retrospective cohort study assessed real‐world diagnostic workups of adult patients suspected of having AL amyloidosis, wild‐type ATTR‐CM (ATTRwt‐CM), or both (AL amyloidosis + ATTRwt‐CM).

**Methods:**

Data were extracted from a large electronic health record and integrated claims‐clinical database (January 2017–June 2023). Workups within 24 months before the first recorded diagnosis were assessed in cohorts with AL amyloidosis (International Classification of Diseases, Tenth Revision code: E85.81 only), ATTRwt‐CM (E85.82 only), or AL amyloidosis + ATTRwt‐CM (E85.81 and E85.82).

**Results:**

Of 1653, 1055, and 59 patients in the AL amyloidosis, ATTRwt‐CM, and AL amyloidosis + ATTRwt‐CM cohorts, respectively, 53%, 61%, and 66% received any type of AL amyloidosis or ATTRwt‐CM test. Across respective cohorts, 42%, 40%, and 49% received an AL amyloidosis workup, and 17%, 15%, and 17% underwent complete monoclonal protein testing (MPT) for AL amyloidosis assessment. In the ATTRwt‐CM and AL amyloidosis + ATTRwt‐CM cohorts, 50% and 46% received an ATTRwt‐CM workup, and 9% and 5% underwent complete MPT before or ≤ 7 days after ^99m^technetium‐pyrophosphate scintigraphy. Diagnostic workups were commonly done by cardiac specialists (≥ 34%) and general medicine providers (≥ 29%).

**Conclusions:**

Notable proportions of patients suspected of having AL amyloidosis, ATTRwt‐CM, and AL amyloidosis + ATTRwt‐CM did not undergo adequate guideline‐recommended diagnostic testing. Due to clinical urgency, improving disease and diagnostic awareness among clinicians is necessary for early, accurate diagnosis and treatment.

**Trial Registration:**

The authors have confirmed clinical trial registration is not needed for this submission.

## 1 Introduction

Cardiac amyloidosis (CA) is a progressive disease characterized by protein misfolding and abnormal deposition of amyloid fibrils throughout the heart. It is an underdiagnosed cause of restrictive cardiomyopathy and heart failure (HF) [1, 2, 3]. The two major types of CA are immunoglobulin light chain (AL) and transthyretin (TTR) amyloidosis.

**FIGURE 1:**
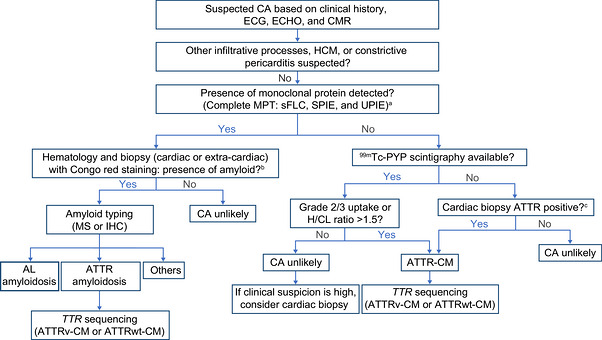
Diagnostic pathway for AL and ATTR‐CM. ^99m^Tc‐PYP, 99m‐technetium pyrophosphate; AL, light chain; ATTRwt‐CM, transthyretin amyloid cardiomyopathy; ATTRv‐CM, variant transthyretin amyloid cardiomyopathy; CA, cardiac amyloidosis; CMR, cardiac magnetic resonance imaging; ECHO, echocardiogram; EKG, electrocardiogram; H/CL, heart to contralateral lung; HCM, hypertrophic cardiomyopathy; IHC, immunohistochemistry; MPT, monoclonal protein testing; MS, mass spectrometry; sFLC, serum free light chain assay; SPIE, serum protein electrophoresis with immunofixation; *TTR*, transthyretin; UPIE, urine protein electrophoresis with immunofixation. ^a^MPT to rule out AL amyloidosis includes sFLC assay (κ/λ ratio < 0.26 or > 1.65), SIFE, and UIFE. SPIE and UPIE consist of two diagnostic tests—SPEP and SIFE, and UPEP (24‐h urine collection for total protein) and UIFE, respectively. In this study, complete MPT consisted of sFLC + SPIE + UPIE. ^b^Positive Congo red staining indicates amyloid deposition in tissues. ^c^Positive Congo red staining and amyloid typing indicate the presence of ATTR. MS‐based method is the gold standard for amyloid typing.

AL amyloidosis is a multi‐systemic disease caused by clonal B or plasma cells producing amyloidogenic monoclonal light chains (lambda or kappa subtype) [4, 5]. Clinical manifestations include HF with preserved ejection fraction (HFpEF), kidney or liver dysfunction, and peripheral or autonomic neuropathy, among others [6, 7, 8]. A suspicion of CA warrants full monoclonal protein testing (MPT) in both serum and urine to detect the presence of monoclonal paraprotein (Figure 1) [9, 10, 11]. Consensus guideline‐recommended complete MPT includes serum free light chain assay (sFLC), serum immunofixation electrophoresis (SIFE), and urine immunofixation electrophoresis (UIFE; 24‐h urine collection), which has ∼99% sensitivity for detecting AL amyloid [9, 10, 11, 12, 13]. Positive results in an MPT require confirmation of amyloid deposits in tissues. Biopsy of surrogate sites (e.g., bone marrow [BM], fat aspirate [FA]) with Congo red staining could detect AL amyloid in ≥ 85% of patients [5, 11]. However, direct biopsy of the affected organ (e.g., heart, kidney, liver) might be needed if the findings from surrogate sites are negative or inconclusive, and clinical suspicion exists for other target organs. Subsequently, mass spectrometry (gold standard) or immunohistochemistry‐based typing is conducted to identify the amyloidogenic protein.

Transthyretin amyloid cardiomyopathy (ATTR‐CM) is caused by the deposition of TTR amyloid in the myocardium. It has two forms: wild‐type ATTR‐CM (ATTRwt‐CM) and hereditary variant ATTR‐CM (ATTRv‐CM), which arises as a result of mutations or variants in the *TTR* gene [14, 15]. Clinical symptoms of ATTR‐CM may include HF, bilateral carpal tunnel syndrome, atrial or ventricular arrhythmias, spinal stenosis, and polyneuropathy. A non‐invasive approach to establish ATTR‐CM diagnosis involves cardiac imaging with bone scintigraphy (e.g., 99m‐technetium pyrophosphate [^99m^Tc‐PYP], ^99m^Tc‐labeled 3,3‐diphosphono‐1,2‐propanodicarboxylic acid, ^99mTc^‐labeled hydroxymethylene diphosphonate), and complete MPT to eliminate the possibility of AL amyloidosis (Figure 1) [9, 11, 14]. A Grade 2/3 uptake in the ^99m^Tc‐PYP scan with normal complete MPT results can confirm an ATTR‐CM diagnosis with 100% specificity [15]. However, an endomyocardial biopsy is recommended if results from the ^99m^Tc‐PYP scintigraphy are inconclusive and monoclonal proteins are present. Moreover, in rare cases, ATTR‐CM and AL amyloidosis or other unrelated monoclonal gammopathy can coexist in a patient [9, 11, 15, 16].

Despite similar manifestations, prognosis and treatment pathways are distinct for patients with AL amyloidosis and ATTRwt‐CM. Diagnostic delays negatively impact patient outcomes, especially for patients with AL amyloidosis who have worse prognosis than those with ATTR‐CM [17, 18]. Untreated patients with AL amyloidosis and ATTRwt‐CM have a median survival of ≤ 1 year and ≤ 5 years, respectively [19, 20, 21, 22]. Previous studies reported that many patients with AL amyloidosis were diagnosed after ≥ 6 months from the onset of initial symptoms and after visiting up to five or more physicians, including primary care providers, cardiologists, hematologists, and oncologists [23, 24, 25, 26].

Early and accurate diagnosis is critical to administer proper treatment to reduce amyloid tissue deposition and progression of amyloid‐related systemic syndromes. This study aimed to assess diagnostic workup patterns of patients suspected of having AL amyloidosis, ATTRwt‐CM, or both AL amyloidosis and ATTRwt‐CM in the United States using real‐world data.

## 2 Methods

### 2.1 Study Design and Patients

**FIGURE 2:**
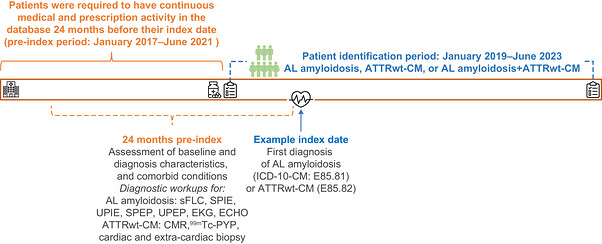
Study design. SPIE includes both SPEP and SIFE. UPIE includes both UPEP and UIFE. ^99m^Tc‐PYP, 99m‐technetium pyrophosphate; AL, light chain; ATTRwt‐CM, wild‐type transthyretin amyloid cardiomyopathy; CMR, cardiac magnetic resonance imaging; ECHO, echocardiogram; EKG, electrocardiogram; ICD‐10‐CM, International Classification of Diseases, Tenth Revision, Clinical Modification system; sFLC, serum free light chain assay; SPEP, serum protein electrophoresis; SPIE, serum protein electrophoresis with immunofixation; UPEP, urine protein electrophoresis; UPIE, urine protein electrophoresis with immunofixation.

This is a retrospective, observational cohort study of patients with International Classification of Diseases, Tenth Revision, Clinical Modification system (ICD‐10‐CM) diagnosis codes for AL amyloidosis, ATTRwt‐CM, or both (AL amyloidosis + ATTRwt‐CM) in the US Optum de‐identified Electronic Health Record dataset (Optum EHR) and Integrated Claims‐Clinical database (Figure 2). Optum EHR is a longitudinal electronic health record repository derived from dozens of healthcare provider (HCP) organizations in the United States across the continuum of care. Patients were indexed between January 1, 2019 and June 30, 2023, at the date of their first AL amyloidosis or ATTRwt‐CM diagnosis. Diagnostic workups in the 24‐months pre‐index period were assessed in mutually exclusive patient cohorts with AL amyloidosis (ICD‐10‐CM: E85.81, not E85.82), ATTRwt‐CM (ICD‐10‐CM: E85.82, not E85.81), or AL amyloidosis + ATTRwt‐CM (both ICD‐10‐CM codes) (Figure 2). Eligible patients were aged ≥ 18 years on the index date with ≥ 24 months of activity and ≥ 1 ICD‐10‐CM code for HF (I50) or cardiomyopathy (I42, I43) in the database during the pre‐index period. Patients with concurrent multiple myeloma or monoclonal gammopathy who were evaluated for suspected AL amyloidosis or ATTRwt‑CM were also included.

### 2.2 Diagnostic Workups

In this study, the types of diagnostic tests for AL amyloidosis included sFLC, serum protein electrophoresis with immunofixation (SPIE), urine protein electrophoresis with immunofixation (UPIE) with 24‐h urine collection, serum protein electrophoresis (SPEP), urine protein electrophoresis (UPEP), electrocardiogram (EKG), and echocardiogram (ECHO). All recorded UPEP and UIFE tests were included in the analysis, regardless of whether they were obtained from 24‐h urine collections or random (spot) samples. Complete MPT consisted of sFLC, SPIE, and UPIE. The types of diagnostic tests for ATTRwt‐CM included cardiac magnetic resonance imaging (CMR), ^99m^Tc‐PYP scintigraphy, and cardiac and extra‐cardiac biopsy (Figure 2). HCP specialties (Table S1) and hospital characteristics (integrated delivery network [IDN] vs. non‐IDN) associated with the diagnostic workups were also assessed. An IDN refers to a healthcare organization or system that operates a network of facilities that may include clinics, hospitals, physician groups, laboratories, and imaging centers providing a continuum of care. IDNs can be academic or community‐based. Academic IDNs are affiliated with academic medical centers or schools that provide clinical care (especially for complex conditions), education and training for students and HCPs, and conduct research [27, 28]. Community IDNs are non‐academic providers affiliated with community hospitals or physicians, serving as the main sites for primary and routine medical care in most communities [27, 28].

### 2.3 Statistical Analysis

All analyses were descriptive and exploratory. Diagnoses were coded using the ICD‐10‐CM system. Procedures were coded using Current Procedural Terminology, fourth edition (CPT); Healthcare Common Procedure Coding System; and ICD‐10‐Procedural Classification System (ICD‐10‐PCS) (Table S2). Prescription drugs were coded using the National Drug Code. Categorical variables were presented as counts and percentages. Continuous variables were shown as counts, means and standard deviations, or medians and ranges. Only non‐missing data were analyzed without imputations. Analyses were performed using SAS software version 9.4 or later (SAS Institute Inc., Cary, NC, USA).

## 3 Results

### 3.1 Baseline Demographics and Characteristics

Of 56.9 million enrolled patients in the Optum EHR database between January 2019 and June 2023, 1653, 1055, and 59 met criteria for the AL amyloidosis, ATTRwt‐CM, and AL amyloidosis + ATTRwt‐CM cohorts, respectively (Table 1, Figure S1). Among the AL amyloidosis, ATTRwt‐CM, and AL amyloidosis + ATTRwt‐CM groups, respectively, most patients were aged ≥ 65 years (66%, 90%, and 81%), of White race (74%, 73%, 58%), enrolled in Medicare (58%, 78%, 78%); and received care from an IDN hospital or clinic (91%, 97%, 97%). Patients with AL amyloidosis were numerically younger versus those with ATTRwt‐CM or AL amyloidosis + ATTRwt‐CM (mean age: 68 vs. 77 or 75 years). A numerically greater proportion of patients in the ATTRwt‐CM and AL amyloidosis + ATTRwt‐CM cohorts versus those in the AL amyloidosis cohort were male (76% and 80% vs. 54%) and resided in the Northeast (43% and 44% vs. 28%).

**TABLE 1:** Baseline demographics and characteristics.

		**Study cohorts**	
	**AL amyloidosis**	**ATTRwt‐CM**	**AL amyloidosis + ATTRwt‐CM**
***n* (%)** **^a^**	***n* = 1653**	***n* = 1055**	***n* = 59**
Age, years			
Mean (SD)	68.29 (11.6)	76.6 (10.5)	74.6 (11.1)
Median (range)	70.0 (19.0–89.0)	79.0 (25.0–89.0)	78.0 (47.0–88.0)
≥ 65 years at index date	561 (33.9)	105 (10.0)	11 (18.6)
< 65 years at index date	1092 (66.1)	950 (90.0)	48 (81.4)
Sex			
Male	893 (54.0)	802 (76.0)	47 (79.7)
Female	758 (45.9)	253 (24.0)	12 (20.3)
Unknown	2 (0.1)	0	0
Race			
White	1216 (73.6)	775 (73.5)	34 (57.6)
Black	288 (17.4)	226 (21.4)	22 (37.3)
Asian	31 (1.9)	6 (0.6)	0
Other/unknown	118 (7.1)	48 (4.5)	3 (5.1)
US region			
Midwest	667 (40.4)	349 (33.1)	22 (37.3)
Northeast	465 (28.1)	453 (42.9)	26 (44.1)
West	237 (14.3)	87 (8.2)	7 (11.9)
South	223 (13.5)	125 (11.8)	≤ 10 (≤ 16.9)^b^
Other/unknown	61 (3.7)	41 (3.9)	0
Insurance			
Medicare	957 (57.9)	825 (78.2)	46 (78.0)
Commercial	931 (56.3)	571 (54.1)	27 (45.8)
Medicaid	166 (10.0)	51 (4.8)	< 5 (< 8.5)^b^
Other	84 (5.1)	45 (4.3)	< 5 (8.5)^b^
Uninsured	110 (6.7)	57 (5.4)	< 5 (< 8.5)^b^
Unknown	237 (14.3)	95 (9.0)	6 (10.2)
Treated at an IDN^c^	1502 (90.9)	1021 (96.8)	57 (96.6)
Index date, y			
2019	435 (26.3)	198 (18.8)	17 (28.8)
2020	390 (23.6)	249 (23.6)	15 (25.4)
2021	323 (19.5)	205 (19.4)	9 (15.3)
2022	318 (19.2)	213 (20.2)	12 (20.3)
2023^d^	187 (11.3)	190 (18.0)	6 (10.2)

Abbreviations: AL, light chain; ATTRwt‐CM, wild‐type transthyretin amyloid cardiomyopathy; IDN, integrated delivery network.

^a^ Values are *n* (%) unless indicated otherwise.

^b^ Data were redacted to reduce the risk of re‐identification due to a small patient number.

^c^ IDN refers to a healthcare system operating as a network of facilities, including clinics, hospitals, physician groups, laboratories, and imaging centers.

^d^ The data for 2023 only cover Quarters 1 and 2.

Across AL amyloidosis, ATTRwt‐CM, and AL amyloidosis + ATTRwt‐CM cohorts, respectively, the most prevalent comorbidities during the 24‐months pre‐index period were hypertensive diseases (61%, 78%, 76%), HF (31%, 68%, 68%) including HFpEF (20%, 47%, 49%), and cardiac arrhythmias (29%, 67%, 63%) (Table 2). Other common comorbid conditions included chronic kidney disease (35.0%) and ischemic heart disease (IHD, 33%) in the AL amyloidosis cohort; CM (63%) and IHD (55%) in the ATTRwt‐CM cohort; and CM (54%), IHD (49%), and diastolic dysfunction (49%) in the AL amyloidosis + ATTRwt‐CM cohort. Moreover, a numerically higher proportion of patients with AL amyloidosis had multiple myeloma (20% vs. 2% with ATTRwt‐CM or 3% with AL amyloidosis + ATTRwt‐CM) and monoclonal gammopathy (20% vs. 5% or 15%).

**TABLE 2:** Summary of baseline comorbidities in the 24 months prior to index date.

		**Study cohorts**	
	**AL amyloidosis**	**ATTRwt‐CM**	**AL amyloidosis + ATTRwt‐CM**
***n* (%)**	***n* = 1653**	***n* = 1055**	***n* = 59**
Cardiovascular disorders			
Heart failure	506 (30.6)	721 (68.3)	40 (67.8)
HFpEF	329 (19.9)	497 (47.1)	29 (49.2)
HFrEF	186 (11.3)	356 (33.7)	22 (37.3)
LVH (or increased wall thickness)	300 (18.1)	453 (42.9)	20 (33.9)
Cardiomyopathy	320 (19.4)	669 (63.4)	32 (54.2)
Cardiac arrhythmias	482 (29.2)	710 (67.3)	37 (62.7)
Cardiac arrest	12 (0.7)	8 (0.8)	0
Paroxysmal tachycardia	122 (7.4)	224 (21.2)	16 (27.1)
Atrial fibrillation and flutter	297 (18.0)	578 (54.8)	30 (50.8)
Other cardiac arrhythmias	264 (16.0)	373 (35.4)	19 (32.2)
Conduction disorders	255 (15.4)	416 (39.4)	15 (25.4)
Atrioventricular and left bundle‐branch block	163 (9.9)	316 (30.0)	11 (18.6)
Other conduction disorders	152 (9.2)	250 (23.7)	13 (22.0)
Pacemaker or ICD	106 (6.4)	224 (21.2)	8 (13.6)
Pacemaker	84 (5.1)	177 (16.8)	7 (11.9)
ICD	49 (3.0)	111 (10.5)	5 (8.5)
Hypertensive diseases	1008 (61.0)	819 (77.6)	45 (76.3)
Diabetes	393 (23.8)	270 (25.6)	17 (28.8)
Ischemic heart disease	541 (32.7)	580 (55.0)	29 (49.2)
Pulmonary embolism	46 (2.8)	41 (3.9)	< 5 (< 8.5)^a^
Pericarditis	116 (7.0)	108 (10.2)	8 (13.6)
Aortic stenosis	177 (10.7)	293 (27.8)	14 (23.7)
Cerebrovascular disease	173 (10.5)	167 (15.8)	10 (16.9)
Peripheral vascular disease	137 (8.3)	114 (10.8)	5 (8.5)
Venous thrombosis	89 (5.4)	61 (5.8)	< 5 (< 8.5)^a^
Heart transplant	< 5 (< 0.3)^a^	11 (1.0)	0
Orthostatic hypotension	86 (5.2)	60 (5.7)	< 5 (< 8.5)^a^
Diastolic dysfunction	329 (19.9)	497 (47.1)	29 (49.2)
Respiratory system disorders			
Pleural effusion	234 (14.2)	219 (20.8)	11 (18.6)
Nervous system disorders			
Carpal tunnel syndrome	73 (4.4)	113 (10.7)	5 (8.5)
Lumbar spinal stenosis	105 (6.4)	129 (12.2)	7 (11.9)
Peripheral neuropathy	252 (15.2)	162 (15.4)	11 (18.6)
Autonomic neuropathy	22 (1.3)	12 (1.1)	0
Paresthesia	89 (5.4)	62 (5.9)	< 5 (< 8.5)^a^
Serious hepatic events			
Acute hepatic failure	67 (4.1)	40 (3.8)	< 5 (< 8.5)^a^
Chronic hepatitis	5 (0.3)	1 (0.1)	0
Cirrhosis	172 (10.4)	84 (8.0)	< 5 (< 8.5)^a^
Hepatic transplant	7 (0.4)	5 (0.5)	0
Jaundice idiopathic	87 (5.3)	81 (7.7)	6 (10.2)
Portal vein thrombosis	81 (4.9)	49 (4.6)	< 5 (< 8.5)^a^
Musculoskeletal and connective tissue disorders			
Atraumatic Achilles tendon rupture	< 5 (< 0.3)^a^	0	0
Atraumatic biceps tendon rupture	0	< 5 (< 0.5)^a^	0
Muscle weakness	61 (3.7)	32 (3.0)	< 5 (< 8.5)^a^
Genitourinary system disorders			
Erectile dysfunction	75 (4.5)	42 (4.0)	6 (10.2)
Testicular dysfunction	19 (1.1)	10 (0.9)	< 5 (< 8.5)^a^
Ovarian dysfunction	7 (0.4)	< 5 (< 0.5)^a^	0
Renal system disorders			
Chronic kidney disease	582 (35.2)	408 (38.7)	27 (45.8)
Acute kidney failure	379 (22.9)	261 (24.7)	19 (32.2)
Proteinuria	289 (17.5)	48 (4.5)	9 (15.3)
Nephrotic syndrome	93 (5.6)	< 5 (< 0.5)^a^	< 5 (< 8.5)^a^
Eye disorders			
Cataracts	112 (6.8)	73 (6.9)	5 (8.5)
Glaucoma	63 (3.8)	65 (6.2)	< 5 (< 8.5)^a^
Vitreous opacities	31 (1.9)	19 (1.8)	< 5 (< 8.5)^a^
Lymphatic disorders			
Edema	390 (23.6)	297 (28.2)	24 (40.7)
Ascites	57 (3.4)	50 (4.7)	5 (8.5)
Splenomegaly	23 (1.4)	13 (1.2)	0
Lymphadenopathy	109 (6.6)	42 (4.0)	< 5 (< 8.5)^a^
Macroglossia	7 (0.4)	0	0
Periorbital purpura	153 (9.3)	134 (12.7)	< 5 (< 8.5)^a^
Sweating disturbances	13 (0.8)	7 (0.7)	0
Other comorbidities			
Diarrhea	175 (10.6)	74 (7.0)	6 (10.2)
Nausea and vomiting	238 (14.4)	90 (8.5)	9 (15.3)
Constipation	213 (12.9)	147 (13.9)	10 (16.9)
Early satiety	13 (0.8)	11 (1.0)	< 5 (< 8.5)^a^
Multiple myeloma	337 (20.4)	21 (2.0)	< 5 (< 8.5)^a^
Monoclonal gammopathy	337 (20.4)	55 (5.2)	9 (15.3)

Abbreviations: AL, light chain; ATTRwt‐CM, wild‐type transthyretin amyloid cardiomyopathy; HFpEF, heart failure with preserved ejection fraction; HFrEF, heart failure with reduced ejection fraction; ICD, implantable cardioverter‐defibrillator; LVH, left ventricular hypertrophy.

^a^ Data were redacted to reduce the risk of re‐identification due to a small patient number.

### 3.2 Diagnostic Workups

Any type of diagnostic test for AL amyloidosis or ATTRwt‐CM was performed in only 53% (AL amyloidosis), 61% (ATTRwt‐CM), and 66% (AL amyloidosis + ATTRwt‐CM) of patients within 24 months before their index date (Table 3).

**TABLE 3:** Diagnostic workups, provider, and hospital characteristics associated with AL amyloidosis.

		**Study cohorts**	
	**AL amyloidosis**	**ATTRwt‐CM**	**AL amyloidosis + ATTRwt‐CM**
***n* (%)**	***n* = 1653**	***n* = 1055**	***n* = 59**
Types of diagnostic workup			
Any	870 (52.6)	643 (61.0)	39 (66.1)
AL only^a^	694 (42.0)	424 (40.2)	29 (49.2)
ATTRwt‐CM only^b^	496 (30.0)	526 (49.9)	27 (45.8)
AL + ATTRwt‐CM	320 (19.4)	307 (29.1)	17 (28.8)
None	783 (47.4)	412 (39.1)	20 (33.9)
Combination of AL diagnostic workup^a^			
Complete MPT (sFLC + SPIE + UFIE)	274 (16.6)	162 (15.4)	10 (16.9)
Complete MPT + ECHO	177 (10.7)	151 (14.3)	9 (15.3)
Complete MPT + EKG	206 (12.5)	155 (14.7)	8 (13.6)
Complete MPT + EKG + ECHO	160 (9.7)	149 (14.1)	8 (13.6)
EKG+ECHO only	601 (36.4)	701 (66.4)	38 (64.4)
Type of hospital for any AL diagnostic workup			
IDN	1078 (65.2)	856 (81.1)	51 (86.4)
Non‐IDN/missing	575 (34.8)	199 (18.9)	8 (13.6)
Provider specialties (any AL diagnostic workup ≥ 1)^b^			
Cardiac specialists	673 (40.7)	787 (74.6)	44 (74.6)
Extra‐cardiac specialists	374 (22.6)	241 (22.8)	22 (37.3)
General medicine	795 (48.1)	656 (62.2)	39 (66.1)
Advanced practitioner	490 (29.6)	347 (32.9)	22 (37.3)
Unknown	377 (22.8)	269 (25.5)	8 (13.6)

Abbreviations: AL, light chain; ATTRwt‐CM, wild‐type transthyretin amyloid cardiomyopathy; ECHO, echocardiogram; EKG, electrocardiogram; IDN, integrated delivery network; sFLC, serum free light chain assay; SPEP, serum protein electrophoresis; SPIE, serum protein electrophoresis with immunofixation; uFLC, urine‐free light chain assay; UPEP, urine protein electrophoresis; UPIE, urine protein electrophoresis with immunofixation.

^a^ The types of AL diagnostic workups included sFLC, SPIE, UPIE, SPEP, UPEP, uFLC, EKG, and ECHO. In this study, complete MPT included sFLC + SPIE + UPIE.

^b^ HCPs were grouped based on specialties as listed in Table S1.

### 3.3 AL Amyloidosis Workups

**FIGURE 3:**
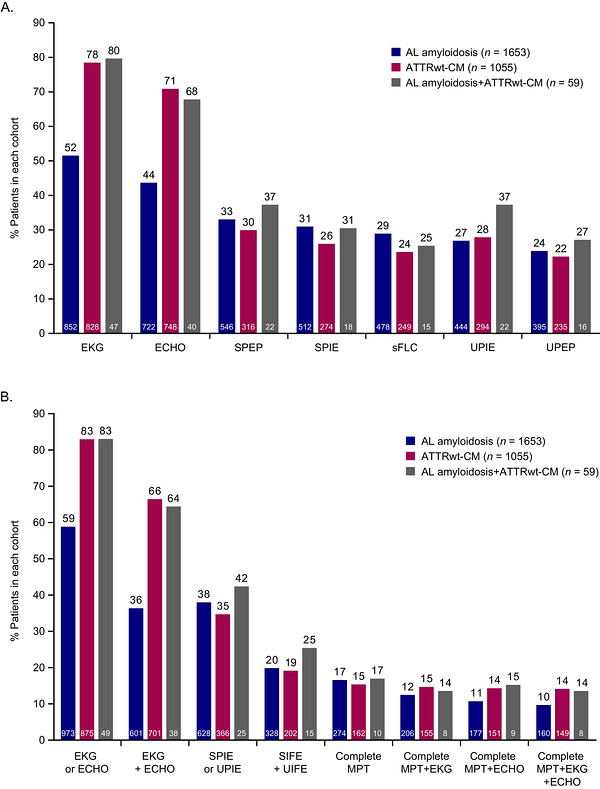
Types (A) and (B) combinations of AL diagnostic workups. Values at the bottom of each bar indicate the actual number of patients. SPIE includes both SPEP and SIFE, whereas UPIE includes both UPEP and UIFE. In this study, complete MPT included sFLC + SPIE + UPIE. AL, light chain; ATTRwt‐CM, wild‐type transthyretin amyloid cardiomyopathy; ECHO, echocardiogram; EKG, electrocardiogram; sFLC, serum free light chain assay; SIFE, serum immunofixation electrophoresis; SPEP, serum protein electrophoresis; SPIE, serum protein electrophoresis with immunofixation; UIFE, urine immunofixation electrophoresis; UPEP, urine protein electrophoresis; UPIE, urine protein electrophoresis with immunofixation.

Overall, fewer than half of the patients had any workup done for AL amyloidosis (42% AL amyloidosis [*n* = 694/1653], 40% ATTRwt‐CM [*n* = 424/1055], and 49% AL amyloidosis + ATTRwt‐CM [*n* = 29/59]) (Table 3). SFLC, SPIE, UPIE, UPEP, and SPEP were performed in 24%–33%, 22%–30%, and 25%–37% of patients in the AL amyloidosis, ATTRwt‐CM, and AL amyloidosis + ATTRwt‐CM cohorts, respectively, and 44%–52%, 71%–78%, and 68%–80% had an EKG or ECHO (Figure 3A). Notably, complete MPT (sFLC + SPIE + UPIE) was conducted in only 17% (AL amyloidosis), 15% (ATTRwt‐CM), and 17% (AL amyloidosis + ATTRwt‐CM) of patients. Respective proportions of patients with complete MPT + ECHO + EKG were 10%, 14%, and 14% (Table 3, Figure 3B). Moreover, although the urine‐free AL test is not part of the diagnostic pathway for AL amyloidosis, this was performed in 3%, 2%, and 3% of patients in the AL amyloidosis, ATTRwt‐CM, and AL amyloidosis + ATTRwt‐CM cohorts, respectively. Overall, AL amyloidosis workups were frequently associated with cardiac specialists (AL amyloidosis [41%], ATTRwt‐CM [75%], AL amyloidosis + ATTRwt‐CM [75%]) and general medicine providers (48%, 62%, 66%; internal medicine [42%, 56%, 61%]) (Table 3). Across cohorts, AL diagnostic tests were often done at IDN hospitals or clinics (≥ 65%).

### 3.4 ATTRwt‐CM Diagnostic Workups

**FIGURE 4:**
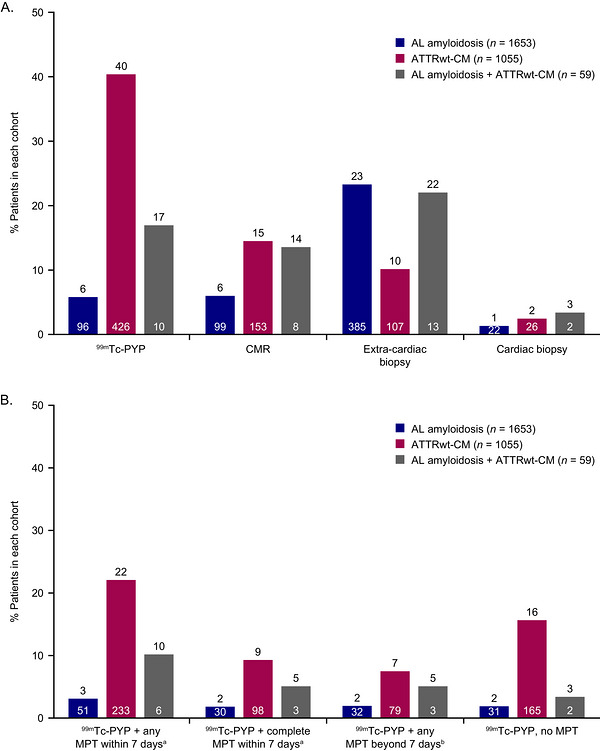
Types of ATTRwt‐CM workups (A) and (B) combinations of ^99m^Tc‐PYP + MPT. Values at the bottom of each bar indicate the actual number of patients. Complete MPT consisted of sFLC + SPIE + UPIE. ^99m^Tc‐PYP, 99m‐technetium pyrophosphate; AL, light chain; ATTRwt‐CM, wild‐type transthyretin amyloid cardiomyopathy; CMR, cardiac magnetic resonance imaging; MPT, monoclonal protein testing. ^a^MPT was performed before or within 7 days after ^99m^Tc‐PYP scan. ^b^MPT was conducted beyond 7 days after ^99m^Tc‐PYP scan.

Among patients in the ATTRwt‐CM and AL amyloidosis + ATTRwt‐CM cohorts, respectively, 50% and 46% had any type of diagnostic test for ATTRwt‐CM. Respective proportions of patients with ^99m^Tc‐PYP scintigraphy were 40% and 17%, whereas 15% and 14% underwent a CMR scan (Figure 4A). Extra‐cardiac biopsy was performed in 10% (ATTRwt‐CM) and 22% (AL amyloidosis + ATTRwt‐CM) of patients (Table S3), whereas 2% and 3% of patients had a cardiac biopsy (Figure 4A). Among patients with a ^99m^Tc‐PYP scan, 9% in the ATTRwt‐CM and 5% in the AL amyloidosis + ATTRwt‐CM cohorts underwent complete MPT before or within 7 days after their PYP scan, whereas 16% and 3% did not get any MPT (Figure 4B). Respective proportions of patients who had combination testing consisting of CMR + ^99m^Tc‐PYP + any type of MPT were 6% and 2%; 15% and 7% had ^99m^Tc‐PYP + any type of MPT (Figure S2). ATTRwt‐CM workups were mostly associated with cardiac specialists (64% [ATTRwt‐CM], 61% [AL amyloidosis + ATTRwt‐CM]) or general medicine providers (42% and 36%), and conducted at IDN hospitals or clinics (73% and 76%) (Table S4).

## 4 Discussion

Findings from this exploratory retrospective study using a large national EHR database showed that notable proportions of patients with AL amyloidosis, ATTRwt‐CM, and AL amyloidosis + ATTRwt‐CM were not worked up and/or diagnosed according to consensus guideline‐recommended pathways. Across cohorts, 47% (AL amyloidosis), 39% (ATTRwt‐CM), and 34% (AL amyloidosis + ATTRwt‐CM) of patients had no relevant workups recorded. Respective proportions of patients who underwent complete MPT were 17%, 15%, and 17%. Moreover, ^99m^Tc‐PYP scintigraphy was performed in 40% (ATTRwt‐CM) and 17% (AL amyloidosis + ATTRwt‐CM) of patients, whereas 9% and 5% underwent complete MPT before or within 7 days after their PYP scan.

Patient characteristics reported in this study are broadly similar to previous reports [14, 29, 30]. Across cohorts, over 50% of patients (median [range] age: 70–79 [19–89] years) were male, of White race, had Medicare insurance, and received care from an IDN hospital or clinic. Consistent with earlier studies, patients with AL amyloidosis manifested comorbidities, including hypertensive diseases (61%) and HF (31%) [29, 31]. Moreover, most patients with ATTRwt‐CM had comorbid conditions, including HF (68%), hypertension (78%), cardiac arrhythmias (67%), and IHD (55%) [32, 33]. Previous studies have reported that patients with multiple myeloma (10%–15%) or monoclonal gammopathy of unknown significance (9%) may develop AL amyloidosis, and up to 30%–38% of patients newly diagnosed with multiple myeloma have AL amyloid deposits [8, 34, 35, 36, 37]. Similarly, in the current study, the proportion of patients with AL amyloidosis and multiple myeloma or monoclonal gammopathy was 20% each.

AL amyloidosis and ATTR‐CM are challenging to diagnose as initial symptoms are often nonspecific, which could lead to misdiagnosis, mistreatment, or delayed appropriate treatment. Prompt and accurate diagnosis is critical, especially for patients with AL amyloidosis, due to poor survival (≤ 1 year without treatment) [19, 38]. This study provides real‐world insights into how patients suspected of having AL amyloidosis and ATTRwt‐CM are diagnosed using a large and diverse cohort derived from EHR data. Overall, general medicine providers and cardiac specialists frequently completed the diagnostic testing for AL amyloidosis (≥ 48% and ≥ 41%) and ATTRwt‐CM (≥ 29% and ≥ 34%), respectively. This is aligned with data reported from real‐world studies in other regions [39]. As notable proportions of the workups were completed outside cardiac specialties, raising disease and diagnostic awareness for CA, AL amyloidosis, and ATTR‐CM in general or non‐cardiology healthcare settings could improve compliance with guideline‐recommended diagnostic pathways. This could potentially contribute to early and improved diagnosis, more prompt treatment initiation, and better patient outcomes [29].

### 4.1 Limitations

This exploratory analysis has several limitations. Analyses based on claims and EHR data have inherent limitations, including potential entry or coding errors, incomplete clinical information, and inconsistencies in data collection and reporting. AL amyloidosis and ATTRwt‐CM are rare diseases that are commonly underreported and underdiagnosed. The ICD‐10 codes used to identify AL amyloidosis (E85.81) and ATTRwt‐CM (E85.82) are relatively new and may be unfamiliar to some HCPs. Furthermore, an ICD‐10 code for AL amyloidosis or ATTRwt‐CM may reflect rule‐out evaluations rather than confirmed clinical diagnoses, contributing to possible misclassification. Moreover, the 24‐month pre‐index period was selected as a reasonable timeframe to capture diagnostic testing patterns due to frequent delays in the diagnosis of AL amyloidosis or ATTRwt‐CM. However, tests conducted > 24 months before the index date or performed outside health systems represented in the Optum dataset would not be captured. These factors, along with other unmeasured confounders, may lead to incomplete characterization of diagnostic workup patterns. This was a descriptive analysis; therefore, no adjustments were made for potential confounding variables. Future studies may help validate our findings by using other real‐world data sources, such as the US Centers for Medicare & Medicaid Services database, which mainly includes patients aged ≥ 65 years. Additional work may also explore restricting analyses to patients with AL amyloidosis or ATTRwt‐CM who later received disease‑specific therapies, to better characterize diagnostic patterns among those with a confirmed diagnosis and subsequent treatment.

## 5 Conclusion

Based on a large national EHR dataset, notable proportions of patients with suspected AL amyloidosis, ATTRwt‐CM, and AL amyloidosis + ATTRwt‐CM were not worked up using guideline‐recommended pathways. Fewer than half of patients across each cohort (34%–47%) had no expected workups recorded as sent or completed. Furthermore, the proportions of patients who did not undergo complete MPT were 83%, 85%, and 83% in the AL amyloidosis, ATTRwt‐CM, and AL amyloidosis + ATTRwt‐CM cohorts, respectively. Increased understanding and education of HCPs, both in specialty and subspecialty branches, on the standard of care and guideline‐recommended diagnostic pathways for AL amyloidosis and ATTR‐CM are needed to provide early and accurate diagnosis for optimal patient outcomes.

## Author Contributions

**Muhamed Baljevic**: conceptualization, writing – review and editing. **Haechung Chung**: conceptualization, methodology, formal analysis, supervision, writing – review and editing. **Jose Alvir**: conceptualization, methodology, supervision, writing – review and editing. **Patrick Feron**: methodology, supervision, writing – review and editing. **Aaron Crowley**: methodology, formal analysis, writing – review and editing. **Darrin Benjumea**: methodology, formal analysis, writing – review and editing. **Yong Chen**: conceptualization, methodology, supervision, writing – review and editing. **Cindi Pankratova**: conceptualization, methodology, supervision, writing – review and editing.

## Funding

This study was sponsored by Pfizer.

## Ethics Statement

Optum EHR data are certified as de‐identified by an independent statistical expert following the US Health Insurance Portability and Accountability Act. Institutional research board approval was not required to conduct this study, as analyses from commercially available de‐identified secondary data sources are considered exempt from the requirements for “human subjects research” [40]. The study was conducted in accordance with Good Practices for Outcomes Research issued by the International Society for Pharmacoeconomics and Outcomes Research.

## Consent

Informed consent from patients was not required as the analysis included anonymized structured data without patient personal information.

## Conflicts of Interest

Muhamed Baljevic reports consultancy for AbbVie and Pfizer; advisory boards for Bristol‐Myers Squibb/Celgene, Janssen Research, Pfizer, Prothena, and Sanofi‐Genzyme; and serving in independent review committees for Parexel. Haechung Chung, Patrick Feron, Yong Chen, and Cindi Pankratova are employees of and hold stock/options in Pfizer. Jose Alvir was an employee of Pfizer at the time of this study. Aaron Crowley and Darrin Benjumea are employees of Genesis Research and are paid consultants to Pfizer in connection with this study.

## Supporting information




**Supporting File 1**: jha270330‐sup‐0001‐FigureS1.pdf

**Supporting File 2**: jha270330‐sup‐0002‐FigureS2.pdf

**Supporting File 3**: jha270330‐sup‐0003‐TableS1.docx

**Supporting File 4**: jha270330‐sup‐0004‐TableS2.docx

**Supporting File 5**: jha270330‐sup‐0005‐TableS3.docx

**Supporting File 6**: jha270330‐sup‐0006‐TableS4.docx

## Data Availability

Research data are confidential due to third‐party contract agreements on data sharing and privacy.
