# Supplementary figures and images for: Real‐World Diagnostic Workup of Patients Suspected for Light Chain Amyloidosis and Wild‐Type Transthyretin Amyloid Cardiomyopathy: A Retrospective Cohort Study Using US Electronic Health Records
Source: EJHaem. 2026 Jun 15;7(3):e70330. doi: 10.1002/jha2.70330 (PMC13267428; doi:10.1002/jha2.70330)

# SUPPLEMENTAL FIGURE S1. Eligible patient selection from the Optum EHR database.

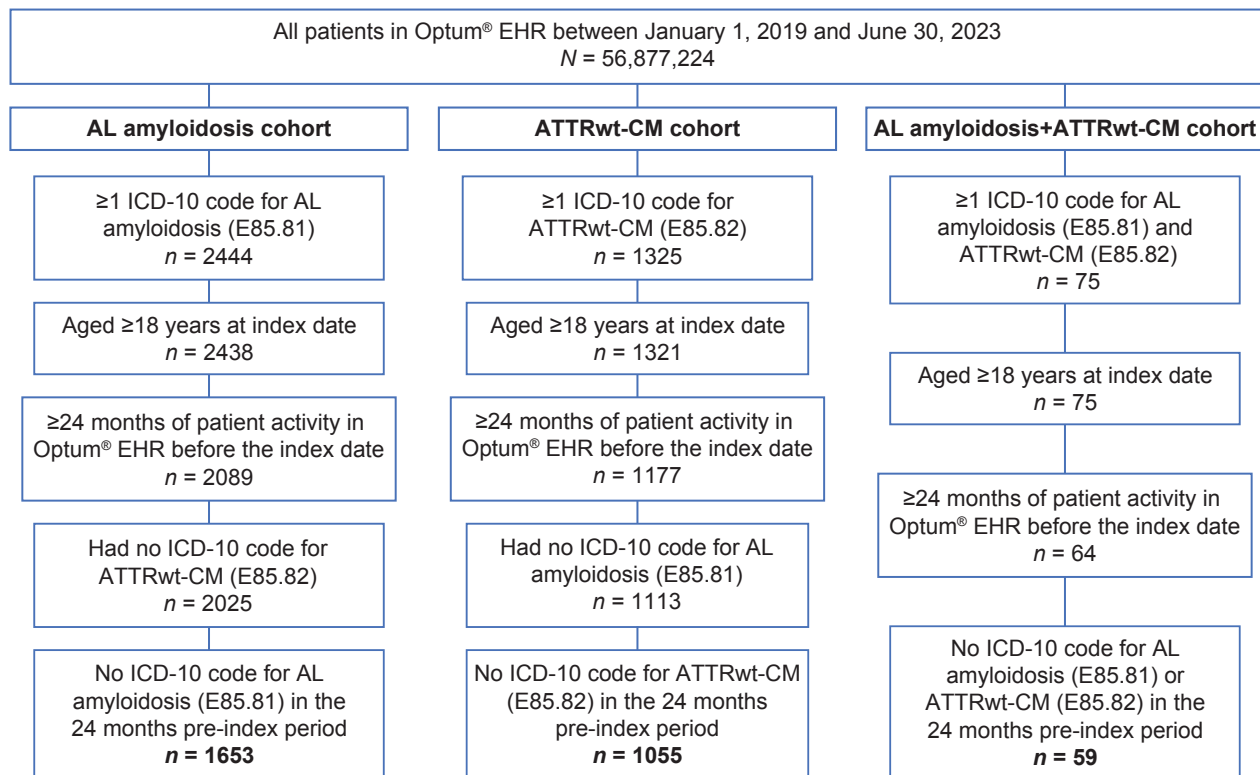

Supplement: Supplementary file 1 — Supporting File 1: jha270330‐sup‐0001‐FigureS1.pdf [file JHA2-7-e70330-s001.pdf]
