# Supplementary material for: Real‐World Diagnostic Workup of Patients Suspected for Light Chain Amyloidosis and Wild‐Type Transthyretin Amyloid Cardiomyopathy: A Retrospective Cohort Study Using US Electronic Health Records
Source: EJHaem. 2026 Jun 15;7(3):e70330. doi: 10.1002/jha2.70330 (PMC13267428; doi:10.1002/jha2.70330)
Supplement: Supplementary file 2 — Supporting File 2: jha270330‐sup‐0002‐FigureS2.pdf [file JHA2-7-e70330-s003.pdf]

SUPPLEMENTAL FIGURE S2. ATTRwt-CM combination of diagnostic workups.

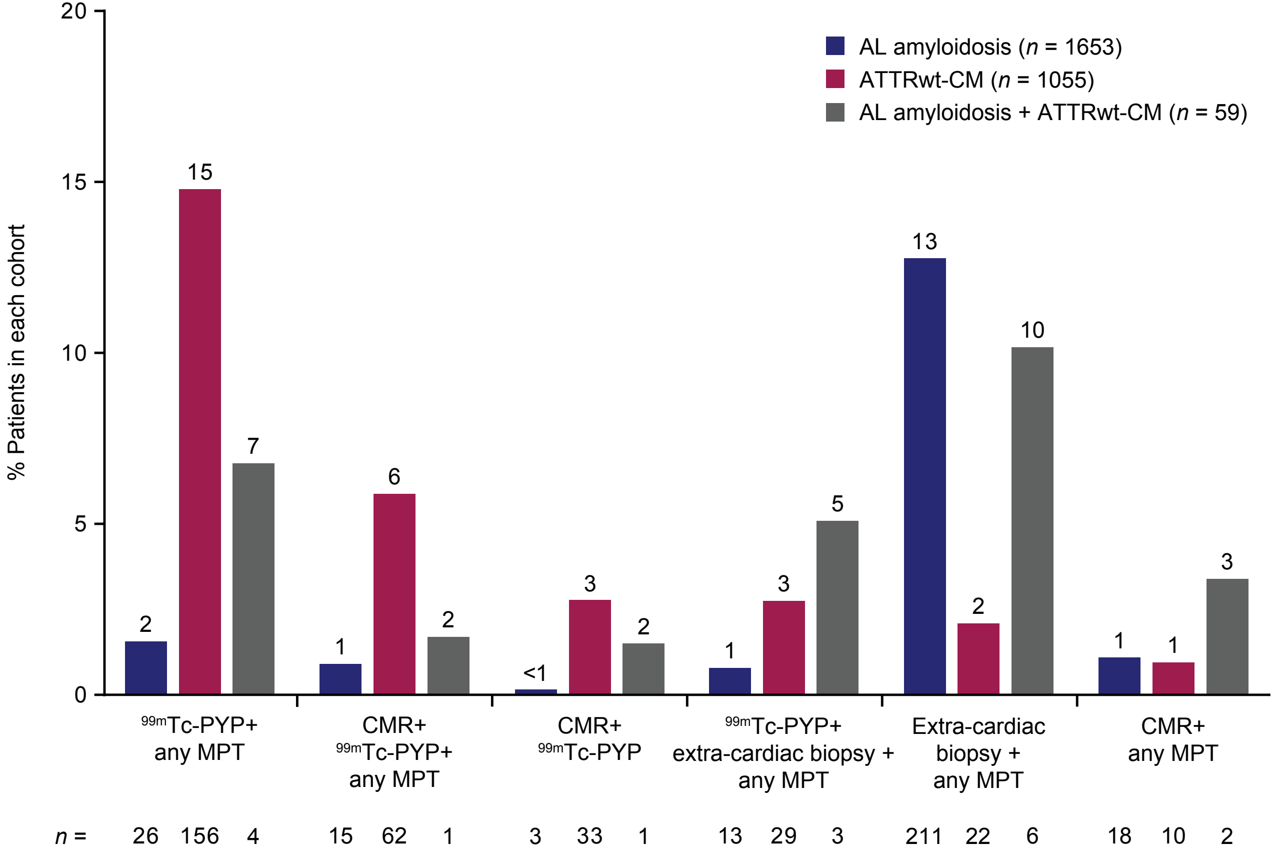

Only combination diagnostic tests performed in ≥3% of patients (in ≥1 cohort) are presented.
Complete MPT included sFLC+SPIE+UPIE.
Extra-cardiac biopsies included bone marrow, fat pad, rectal, buccal, and gastric tissues (Supplemental Table S3).
Abbreviations: ^{99m}Tc-PYP, 99m-Technetium pyrophosphate; AL, light chain; ATTRwt-CM, wild-type transthyretin amyloid cardiomyopathy; CMR, cardiac magnetic resonance imaging; MPT, monoclonal protein testing.
