# Supplementary material for: Real‐World Diagnostic Workup of Patients Suspected for Light Chain Amyloidosis and Wild‐Type Transthyretin Amyloid Cardiomyopathy: A Retrospective Cohort Study Using US Electronic Health Records
Source: EJHaem. 2026 Jun 15;7(3):e70330. doi: 10.1002/jha2.70330 (PMC13267428; doi:10.1002/jha2.70330)
Supplement: Supplementary file 3 — Supporting File 3: jha270330‐sup‐0003‐TableS1.docx [file JHA2-7-e70330-s002.docx]

| **SUPPLEMENTAL TABLE S1** Physician specialty grouping. | |
| --- | --- |
| **Category** | **Specialties** |
| **Cardiac specialties** | Cardiology, cardiac electrophysiology, intensive cardiac rehabilitation, vascular surgery, cardiac surgery, interventional cardiology, and advanced heart failure transplant cardiology |
| **Extra-cardiac specialties** | Gastroenterology, neurology, ophthalmology, orthopedic surgery, diagnostic radiology, urology, nuclear medicine, optometry, hematology, hematology/oncology, medical oncology, surgical oncology, radiation oncology, and interventional radiology |
| **General medicine** | General practice, general surgery, family practice, internal medicine, pediatric medicine, geriatric medicine, nurse practitioner, preventive medicine, emergency medicine, physician assistant, and hospitalist |
| **Advanced practitioners** | Allergy/immunology, otolaryngology, anesthesiology, dermatology, neurosurgery, obstetrics/gynecology, oral surgery (dentists only), pathology, sports medicine, plastic and reconstructive surgery, physical medicine and rehabilitation, psychiatry, geriatric psychiatry, colorectal surgery (formerly proctology), pulmonary disease, thoracic surgery, chiropractic, nephrology, hand surgery, infectious disease, endocrinology, podiatry, psychologist (billing independently), audiologist (billing independently), rheumatology, clinical psychologist, optician, gynecological/oncology, ocularist, and dentist |
