# Supplementary material for: Real‐World Diagnostic Workup of Patients Suspected for Light Chain Amyloidosis and Wild‐Type Transthyretin Amyloid Cardiomyopathy: A Retrospective Cohort Study Using US Electronic Health Records
Source: EJHaem. 2026 Jun 15;7(3):e70330. doi: 10.1002/jha2.70330 (PMC13267428; doi:10.1002/jha2.70330)
Supplement: Supplementary file 4 — Supporting File 4: jha270330‐sup‐0004‐TableS2.docx [file JHA2-7-e70330-s005.docx]

| **SUPPLEMENTAL TABLE S2** CPT or ICD-10-PCS for diagnostic tests. | |
| --- | --- |
| **Diagnostic workup** | **CPT or ICD code** |
| Monoclonal protein testing | CPT  83521 Immunoglobulin light chains (ie, kappa, lambda), free  83883 Serum free light chains  84155 Protein total, Protein electrophoresis  84165 Protein electrophoresis serum  84156 or 84166 Urine protein electrophoresis  86334 Immunofixation electrophoresis; serum  86335 Immunofixation electrophoresis; other fluids with concentrations (eg, urine, CSF)  82784 Gammaglobulin (immunoglobulin); IgA, IgD, IgG, IgM |
| Cardiac magnetic resonance | CPT  75557 Cardiac magnetic resonance imaging for morphology and function without contrast material  75559 Cardiac magnetic resonance imaging for morphology and function without contrast material, with stress imaging  75561 Cardiac magnetic resonance imaging for morphology and function without contrast material(s), followed by contrast material(s) and further sequences  75563 Cardiac magnetic resonance imaging for morphology and function without contrast material(s), followed by contrast material(s) and further sequences, with stress imaging  75565 Cardiac magnetic resonance imaging for velocity flow mapping (List separately in addition to code for primary procedure)  93356 MRI [CPT: 75552–75556] or ECHO [CPT: 93356 + 0399T]  0399T MRI [CPT: 75552–75556] or ECHO [CPT: 93356 + 0399T] |
| ^99m^Tc-pyrophosphate | CPT  78800 Radiopharmaceutical localization of tumor or distribution of radiopharmaceutical agent(s); planar  78803 Radiopharmaceutical localization of tumor or distribution of radiopharmaceutical agent(s); tomographic (SPECT)  78802 Radiopharmaceutical localization of tumor, inflammatory process or distribution of radiopharmaceutical agent(s) (includes vascular flow and blood pool imaging, when performed); planar  A9538 ICD-10-PCS 92.19, 92.18 Technetium tc-99m pyrophosphate, diagnostic |
| Cardiac biopsy | CPT  93505 Endomyocardial biopsy  ICD-10 PCS  02BN0ZX, 02BN3ZX, 02BN4ZX Biopsy of heart lining  02BM3ZX, 02BM4ZX Excision of Ventricular Septum |
| Extra-cardiac biopsy | CPT  10021, 10022 Fat pad biopsy  11106 Incisional biopsy skin single lesion; fat pad biopsy  11104 Punch biopsy skin single lesion; fat pad biopsy  88344 Immunohistochemistry (IHC)  38221, 38222, 38220 Diagnostic bone marrow; biopsy(ies)  45100, 45305, 45331 Rectal biopsy  42400, 42405 Salivary biopsy  40808 Buccal biopsy  43238, 43239, 43242 Gastric tissue biopsy  ICD-10-PCS  0J983ZX Drainage of Abdomen Subcutaneous Tissue and Fascia, Percutaneous Approach, Diagnostic; Fat pad biopsy  0DBP0ZX, 0DBN8ZX, 0DBP8ZX, 0DBP3ZX, 0DBP4ZX, 0DBP7ZX, 0DDP8ZX, 0DDP3ZX, 0DDP4ZX Rectal biopsy  0CBJ0ZX, 0CBJ3ZX, 0CB83ZX, 0CB93ZX, 0CBD3ZX, 0CBF3ZX, 0CBG3ZX, 0CBH3ZX, 0CB80ZX, 0CB90ZX, 0CBD0ZX, 0CBF0ZX, 0CBG0ZX, 0CBH0ZX Salivary biopsy  0CB40ZX, 0CB43ZX, 0CB4XZX Buccal biopsy  07DQ3ZX, 07DR3ZX, 07DS3ZX, 07DT3ZX, 079T3ZX Bone marrow biopsy  0DB68ZX, 0DB78ZX, 0DD68ZX, 0DD78ZX Gastric tissue biopsy |
| Echocardiogram | CPT  93303–93356 Echocardiography procedures  ICD-10-PCS  X2JAX47 Inspection of heart using transthoracic echocardiography, computer-aided guidance, new technology group 7 |
| Electrocardiogram | CPT  93000 Electrocardiogram, routine ECG with at least 12 leads; with interpretation and report  3120F 12-lead ECG performed  0178T Electrocardiogram, 64 leads or greater, with graphic presentation and analysis; with interpretation and report  0179T Electrocardiogram, 64 leads or greater, with graphic presentation and analysis; tracing and graphics only, without interpretation and report  0180T Electrocardiogram, 64 leads or greater, with graphic presentation and analysis; interpretation and report only  93010 Electrocardiogram, routine ECG with at least 12 leads; interpretation and report only  93660 Evaluation of cardiovascular function with tilt table evaluation, with continuous ECG monitoring and intermittent blood pressure monitoring, with or without pharmacological intervention  93268 Electrocardiogram (ECG) up to 30 days continuous with symptom monitoring and review and report by health care professional  93224 Electrocardiogram (ECG) 2-day continuous with review and report by health care professional  93225 Electrocardiogram (ECG) 2-day continuous  93226 Electrocardiogram (ECG) 2-day continuous with report  93227 Electrocardiogram (ECG) 2-day continuous with review by health care professional  93228 Electrocardiogram (ECG) up to 30 days continuous with review and report by health care professional  93229 Electrocardiogram (ECG) up to 30 days continuous with transmission of patient triggered events with review and report by health care professional  93268 Electrocardiogram (ECG) up to 30 days continuous with symptom monitoring and review and report by health care professional  93270 Electrocardiogram (ECG) up to 30 days continuous with symptom monitoring  93271 Electrocardiogram (ECG) up to 30 days continuous with symptom monitoring and transmission and analysis  93272 Electrocardiogram (ECG) up to 30 days continuous with symptom monitoring, transmission and review and report by health care professional  0295T External electrocardiographic recording for more than 48 hours up to 21 days by continuous rhythm recording and storage; includes recording, scanning analysis with report, review and interpretation  0296T External electrocardiographic recording for more than 48 hours up to 21 days by continuous rhythm recording and storage; recording (includes connection and initial recording)  0297T External electrocardiographic recording for more than 48 hours up to 21 days by continuous rhythm recording and storage; scanning analysis with report  0298T External electrocardiographic recording for more than 48 hours up to 21 days by continuous rhythm recording and storage; review and interpretation  ICD-10-PCS  4A020 Cardiac, open |
| Abbreviations: CPT, Current Procedural Terminology; CSF, cerebrospinal fluid; ICD-10, International Classification of Diseases, Tenth Revision ICD-10 PCS, ICD-10-Procedural Classification System; ECHO, echocardiogram; ECG, electrocardiogram; IgA, immunoglobulin A; IgD, immunoglobulin D; IgG, immunoglobulin G; IgM, immunoglobulin; IHC, immunohistochemistry; MRI, magnetic resonance imaging; SPECT, single-photon emission computed tomography. | |
