# Supplementary material for: Real‐World Diagnostic Workup of Patients Suspected for Light Chain Amyloidosis and Wild‐Type Transthyretin Amyloid Cardiomyopathy: A Retrospective Cohort Study Using US Electronic Health Records
Source: EJHaem. 2026 Jun 15;7(3):e70330. doi: 10.1002/jha2.70330 (PMC13267428; doi:10.1002/jha2.70330)
Supplement: Supplementary file 5 — Supporting File 5: jha270330‐sup‐0005‐TableS3.docx [file JHA2-7-e70330-s004.docx]

| **SUPPLEMENTAL TABLE S3** Types of extra-cardiac biopsy included in the ATTRwt-CM diagnostic workup. | | | |
| --- | --- | --- | --- |
|  |  | **Study cohorts** |  |
|  | **AL amyloidosis** | **ATTRwt-CM** | **AL amyloidosis + ATTRwt-CM** |
| **n (%)^a^** | ***n* = 1653** | ***n* = 1055** | ***n* = 59** |
| **Extra-cardiac biopsy (all types)** | 385 (23.3) | 107 (10.1) | 13 (22.0) |
| Bone marrow | 248 (64.4) | 35 (32.7) | 6 (46.2) |
| Gastric | 139 (36.1) | 59 (55.1) | 7 (53.8) |
| Fat pad | 39 (10.1) | 18 (16.8) | <5 (<38.5)^b^ |
| Rectal | 16 (4.2) | 5 (4.7) | 0 |
| Buccal | <5 (<0.3)^b^ | 0 | 0 |
| ^a^n numbers show number of patients for each type of extra-cardiac biopsy. Percentages for all types of extra-cardiac biopsy were calculated using the overall number of patients in the cohort. Percentages for each type were calculated using the overall number of patients who had any type of extra-cardiac biopsy in each cohort. Some patients had ≥1 type of biopsy.  ^b^Data was redacted to reduce the risk of re-identification due to a small patient number.  Abbreviations: AL, light chain; ATTRwt-CM, transthyretin amyloid cardiomyopathy. | | | |
