# Supplementary material for: Real‐World Diagnostic Workup of Patients Suspected for Light Chain Amyloidosis and Wild‐Type Transthyretin Amyloid Cardiomyopathy: A Retrospective Cohort Study Using US Electronic Health Records
Source: EJHaem. 2026 Jun 15;7(3):e70330. doi: 10.1002/jha2.70330 (PMC13267428; doi:10.1002/jha2.70330)
Supplement: Supplementary file 6 — Supporting File 6: jha270330‐sup‐0006‐TableS4.docx [file JHA2-7-e70330-s006.docx]

| **SUPPLEMENTAL TABLE S4** Hospital and provider specialties associated with ATTRwt-CM diagnostic workups. | | | |
| --- | --- | --- | --- |
|  |  | **Study cohorts** |  |
|  | **AL amyloidosis** | **ATTRwt-CM** | **AL amyloidosis + ATTRwt-CM** |
| **n (%)** | ***n* = 1653** | ***n* = 1055** | ***n* = 59** |
| Type of hospital (any ATTRwt-CM diagnostic workup^a^) |  |  |  |
| IDN | 816 (49.4) | 769 (72.9) | 45 (76.3) |
| Non-IDN/Missing | 837 (50.6) | 286 (27.1) | 14 (23.7) |
| Provider specialties (any ATTRwt-CM diagnostic workup)^b^ |  |  |  |
| Cardiac specialists | 566 (34.2) | 677 (64.2) | 36 (61.0) |
| Extra-cardiac specialists | 269 (16.3) | 171 (16.2) | 19 (32.2) |
| General medicine | 474 (28.7) | 442 (41.9) | 21 (35.6) |
| Advanced practitioner | 220 (13.3) | 159 (15.1) | 14 (23.7) |
| Unknown | 258 (15.6) | 238 (22.6) | 13 (22.0) |
| ^a^The types of ATTRwt-CM workup included ^99m^Tc-PYP, CMR, cardiac biopsy, and extra-cardiac biopsy (**Supplemental Table S3**).  ^b^HCPs were grouped based on specialties as listed in **Supplemental Table S1**.  Abbreviations: ^99m^Tc-PYP, 99m-Technetium pyrophosphate; AL, light chain; ATTRwt-CM, transthyretin amyloid cardiomyopathy; CMR, cardiac magnetic resonance imaging; IDN, Integrated Delivery Network. | | | |
